# Moving Complementary Feeding Forward: Report on a Workshop of the Federation of International Societies for Pediatric Gastroenterology, Hepatology and Nutrition (FISPGHAN) and the World Health Organization Regional Office for Europe

**DOI:** 10.1097/MPG.0000000000003562

**Published:** 2022-07-15

**Authors:** Melissa A. Theurich, Mary Fewtrell, Jeannine Baumgartner, Michael R. Perkin, Joao Breda, Kremlin Wickramansinghe, Martin W. Weber, Berthold Koletzko

**Affiliations:** From the * LMU - Ludwig-Maximilians-Universität Munich, Div. Metabolic and Nutritional Medicine, Dept. Pediatrics, Dr von Hauner Children’s Hospital, LMU University Hospitals, Munich, Germany; the † Current address: Institute of Social Medicine and Health Systems Research, Otto von Guericke University Magdeburg, Medical Faculty, Magdeburg, Germany; the ‡ University College London Great Ormond Street Institute of Child Health, London, United Kingdom; the § Laboratory of Human Nutrition, ETH Zurich, Zurich, Switzerland; the ∥ Population Health Research Institute, St George’s, University of London, London, United Kingdom; the ¶ Division of Country Health Policies and Systems, WHO, Athens, Greece; the # WHO Regional Office for Europe, Division of country health Programmes, Copenhagen, Denmark; the ** WHO Regional Office for Europe, Division of Country Health Policies and Systems, Copenhagen, Denmark.

**Keywords:** commercial complementary food, complementary feeding, marketing, nutrition policy, standards

## Abstract

The WHO Regional Office for Europe and the Federation of International Societies for Pediatric Gastroenterology, Hepatology, and Nutrition held a joint workshop, “Moving Complementary Feeding Forward” at the sixth World Congress Pediatric Gastroenterology, Hepatology, and Nutrition in 2021. Here we summarize workshop presentations and discussions. The workshop covered health implications of complementary feeding (CF) including allergies, challenges to meet dietary needs during the CF period, quality of commercial complementary foods (CFD) and respective marketing practices, national CF guidelines in Europe, a nutrient profiling system for CFD, and global policy perspectives on the standards and regulation of marketing for CFD. Adequate CF practices are of critical importance for short and long-term child health, prevention of nutrient deficiencies, normal growth and development, and reducing the risk of allergies. The workshop identified the need to improve feeding practices, harmonize evidence-based information and develop guidance jointly with various stakeholders, improve the composition and marketing practices of commercial CFD and their transparent labeling based on nutrient profiling. Renewed efforts for collaboration between scientists, public health experts, pediatric associations, national governments, and the WHO are necessary for advancing progress.

**What Is Known**

- Guidance on complementary feeding (CF) was last published by PAHO and WHO in 2003 and 2005, respectively.
- Substantial gaps exist between the need for CF guidance and effective action and policies.

**What Is New**

- Evidence shows the unsatisfactory quality of commercial complementary foods (CFD), and inappropriate marketing and promotion.
- Marked heterogeneity exists in national recommendations across Europe on timing and composition of CF, and few national policies exist to control inappropriate marketing and promotion.
- Collaboration of stakeholders should establish and share evidence-informed guidance to promote healthy CF practices and reduce inappropriate composition and marketing.

The World Health Organization (WHO) Regional Office for Europe and the Federation of International Societies for Pediatric Gastroenterology, Hepatology and Nutrition (FISPGHAN) held a joint workshop, “Moving Complementary Feeding Forward” at the 6th World Congress on Pediatric Gastroenterology, Hepatology and Nutrition in June 2021. It addressed health implications of complementary feeding (CF), implementation and policy perspectives.

The WHO Regional Office for Europe is 1 of 6 regional WHO offices. FISPGHAN is a non-profit organization that advocates for child health, promotes research and education related to pediatric gastroenterology, hepatology, and nutrition (PGHN), and organizes the World Congress of PGHN every 4 years for sharing and discussing scientific evidence in the field.

## METHODS

This narrative review summarizes the workshop presentations and discussions from materials shared by the authors and workshop recordings. The workshop was designed and moderated by Martin Weber, Program Manager for Child and Adolescent Health and Development, WHO Regional Office for Europe, and Berthold Koletzko, then FISPGHAN President and Else Kröner Seniorprofessor of Pediatrics at LMU Munich.

## IMPORTANCE OF CF

CF has an important impact on child health, development, and long-term quality of life. The CF period, from about 6–24 months of life, is a critical period when nutrient needs are high due to rapid growth and development. Most childhood undernutrition globally develops during this time period (1). Here we define complementary foods (CFD) as all energy-providing solids and liquids provided other than human milk or infant formula provided in addition to these. CF is needed, in addition to breastmilk or infant formula, to meet the needs for energy and micronutrients (2). For example, considerable amounts of iron accumulate in fetal body stores in utero that are utilized through rapid growth during the first postnatal months. Stores become depleted at around the end of the fifth month of life, and CFD need to provide iron along with other critical nutrients to support adequate nutrient status.

The key goals of the workshop were to review health impacts of CF, current recommendations on CF, the quality of commercial CFD, and respective marketing practices. Opportunities for improvements in CFD, feeding practices, and global policies were explored.

## HEALTH IMPACT OF CF QUALITY – WHAT IS IMPORTANT?

### Mary Fewtrell

CFD are needed from about the middle of the first year of life onwards for both nutritional and non-nutritional reasons. Macro- and micronutrients in CFD complement those provided by breastmilk, when breastmilk alone can no longer meet requirements. Non-nutritional considerations of CF include the transition from a liquid to a family diet, with non-nutritional quantities of food potentially inducing tolerance for allergy prevention and influencing taste and food preferences in later life.

Infancy is a period of rapid growth and brain development when there are high risks for specific nutrient deficiencies, infections, stunting, wasting, and infant death, especially in low-income countries (3). Stunting has long-term implications, including a decrease in the years of schooling, lower cognitive test scores, household income, and increased poverty risk (4,5). Globally, CF interventions are the fourth most important evidence-based intervention, after breastfeeding, for preventing undernutrition (6).

In high-income countries, a major concern is excess energy intake promoting excessive early weight gain with increased later risk of overweight and obesity. In contrast, in low- and middle-income countries a predominant concern is providing sufficient energy-dense foods to prevent growth faltering and undernutrition, especially in the presence of recurrent and chronic infections and inflammation. Many countries undergoing nutritional transition suffer from the double burden of malnutrition, with concomitant occurrence of both undernutrition and obesity. Promotion of appropriate CF is a double-duty action because it addresses both undernutrition and overweight and obesity (7).

Nutrient gaps differ depending on context and the primary milk feeding (human milk, infant formula, or mixed feeding). It is helpful to understand which nutrients are of importance for a particular population. Intakes of alpha-linolenic acid, docosahexaenoic acid (DHA), iron, vitamin D, and iodine are low in many European infants and young children (8). Globally, infants and young children often achieve inadequate intakes of vitamins A, D, B12, C and folate, calcium, iron, iodine, zinc, and DHA (9). Fat intake should not be restricted to ensure sufficient energy density.

Providing adequate protein for infants may be challenging in low-income countries, especially when animal source foods are not available, affordable, or acceptable. High protein intakes may increase the risk of overweight and obesity, with a stronger association for milk protein than for non-milk animal protein (10). Defining appropriate protein intakes is particularly difficult in countries experiencing the double burden of malnutrition.

## CHALLENGES IN MEETING MICRONUTRIENT NEEDS DURING THE CF PERIOD

### Jeannine Baumgartner

Globally, 22% of children under 5 years are stunted (11), and 33% of children in low- and middle-income countries do not meet basic milestones in cognitive or social-emotional development (12), which has marked implications for health, well-being, disease susceptibility and productivity, and ultimately contributes to poverty. Micronutrient deficiencies are a major contributing factor.

**FIGURE 1.**
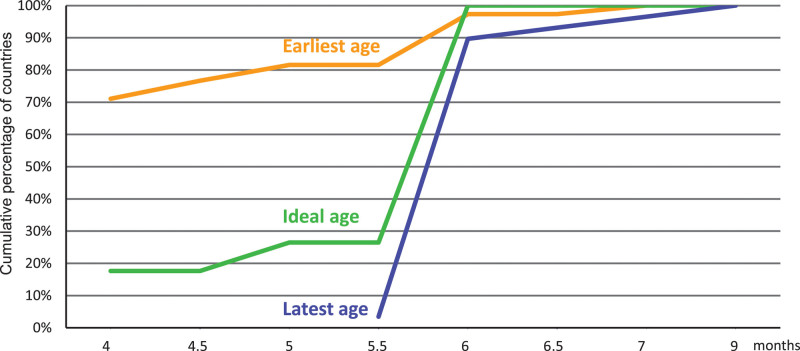
Main causes of micronutrient deficiencies and examples of underlying causes during the complementary feeding period. Modified from reference (13).

Main causes of micronutrient deficiencies include (1) inadequate dietary intake, (2) poor bioavailability, and (3) poor health status. In infants and young children, inadequate dietary intake can be a result of household food insecurity, and/or poor parental feeding practices (Fig. 1) (13). Poor micronutrient bioavailability can result from dietary components inhibiting absorption (e.g. phytic acid and polyphenols), or disease-associated inflammation affecting absorption and utilization. Poor health status may cause micronutrient deficiencies by decreasing appetite, increasing micronutrient losses (e.g. diarrhea), or impairing micronutrient bioavailability. Micronutrient requirements per kilogram of body weight and energy intake are higher during infancy and early childhood than at any other time in life. Thus, CFD need to be micronutrient-dense and diverse.

The WHO and United Nations Children’s Fund recommend that children aged 6–23 months meet minimum dietary diversity. This is defined as daily intake of at least 5 of the following 8 food groups: (1) breastmilk, (2) grains, roots, and tubers, (3) pulses, nuts, and seeds, (4) dairy products, (5) flesh foods, (6) eggs, (7) vitamin A-rich fruits and vegetables, and (8) all other fruits and vegetables (14). Globally, only 29% of infants and young children meet this goal (15).

Strategies to improve micronutrient intakes include promotion of micronutrient-dense and diverse CFD, nutrient supplementation, fortification of commercial CFD, biofortification of staple foods, and fortification of CFD with multiple-micronutrient powders or small-quantity lipid-based nutrient supplements. Each of these strategies has advantages and disadvantages in its sustainability, affordability, accessibility, and efficacy in coverage of key populations.

Main challenges in meeting nutrient requirements include inappropriate feeding practices, monotonous diets dominated by cereal-based porridges, low intake of animal source foods, fruits, and vegetables, and poor bioavailability of micronutrients. Potential interventions include dietary modification through counseling or behavior change communication, food fortification, supplementation, and improved infectious disease prevention and management.

## CF AND ALLERGY RISK

### Michael Perkin

The prevalence of food allergy in Europe appears to be increasing (16). Recent evidence shows early diet to affect the risk of developing food allergy, however, current infant feeding guidelines around the world are heterogeneous.

Du Toit et al (17). reported a lower prevalence of peanut allergy in 4–18-year-old children in Israel (0.17%) compared to the UK (1.85%). Israeli infants and children aged 8–14 months had a higher median intake of 7.1 g of peanut protein per month, compared to 0 g per month in the UK, and they were introduced earlier to peanuts (17).

A randomized trial of peanut consumption or avoidance in 640 four- to 10-month-old infants at risk for peanut allergy showed an 81% reduction in the risk for peanut allergy with peanut consumption in the first year of life (18). A subsequent study demonstrated effectiveness of early introduction of individual allergenic foods in preventing food allergy in subgroups at high risk of food allergy (19).

New national guidelines advocating introduction of allergenic foods mostly before the age of 6 months have resulted in changes in infant feeding practices. Compared with 2007–2011, a 3-fold increase in peanut introduction was observed in Australia following the publication of the new IYCF guidelines (20). Recent European guidelines on preventing the development of food allergy recommend introducing cooked egg into the infant diet (21). In populations with a high prevalence of peanut allergy, introduction of peanuts is recommended, and the age of 4 to 6 months of life is recommended as the most prevention-effective age to introduce egg and peanut (21). Peanuts should be introduced in a safe form that avoids the risk of choking, for example as smooth unsalted peanut butter.

## RECOMMENDATIONS ON CF ACROSS EUROPE – RESULTS OF A SURVEY

### Berthold Koletzko

Available data indicate that current infant and young child feeding (IYCF) practices are often less than satisfactory and deviate from evidence-based guidance and show marked differences (22,23) (Table 1, which may contribute to heterogeneity in IYCF practices).

**TABLE 1.** Selected guidance papers on complementary feeding published during the last 2 decades

2003: PAHO/WHO Guiding principles for complementary feeding of the breastfed child (1)
2003: WHO Regional Office for Europe. Feeding and nutrition of infants and young children (2)
2005: WHO Guiding principles for feeding non-breastfed children 6–24 months of age (3)
2008: Complementary feeding: a commentary by the Committee on Nutrition, European Society for Pediatric Gastroenterology, Hepatology and Nutrition (ESPGHAN) (4)
2009: Scientific Opinion on the appropriate age of introduction of complementary feeding of infants by the European Food Safety Authority (EFSA) (5)
2016: World Health Assembly (WHA) Resolution 69.9 Guidance on ending the inappropriate promotion of foods for infants and young children
2017: WHO Implementation Guidance on Ending The Inappropriate Promotion of Foods for infants and young children (6)
2017: Complementary Feeding: A Position Paper by the ESPGHAN Committee on Nutrition (7)
2019: WHO Regional Office for Europe. Ending inappropriate promotion of commercially available complementary foods for infants and young children between 6 and 36 months in Europe (8)
2019: A discussion paper outlining the first steps in developing a nutrient profile model to drive changes to product composition and labeling and promotion practices in the WHO European Region (9)
2019: European Food Safety Authority (EFSA). Appropriate age range for introduction of complementary feeding into an infant’s diet (10)
2021: European Academy of Allergy and Clinical Immunology (EAACI) guideline: Preventing the development of food allergy in infants and young children (11)

**FIGURE 2.**
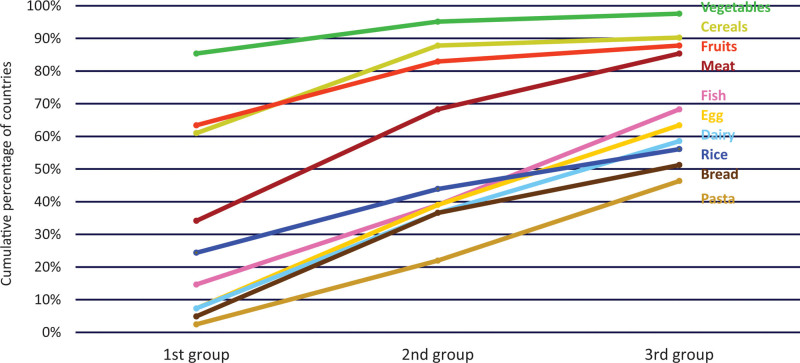
Recommended earliest, ideal and latest ages for introduction of complementary feeding across 43 countries in the WHO European region. Modified from reference (24).

The European Society for Pediatric Gastroenterology, Hepatology and Nutrition (ESPGHAN) and the WHO Regional Office for Europe surveyed national recommendations on IYCF in the WHO European Region, comprising 53 countries (24). The results of 48 of 53 surveyed countries that responded (91%) indicate that national governments lead recommendations on CF in 94% of countries, while of countries professional organizations take responsibility in 9%. Almost all countries (93%) recommend exclusive breastfeeding for the first 6 months of life, but most countries also provided contradicting recommendations on introduction of CF, with 82% recommending introduction of CF before the age of 6 months (mostly from 4 months onwards) (Fig. 2).

**FIGURE 3.**
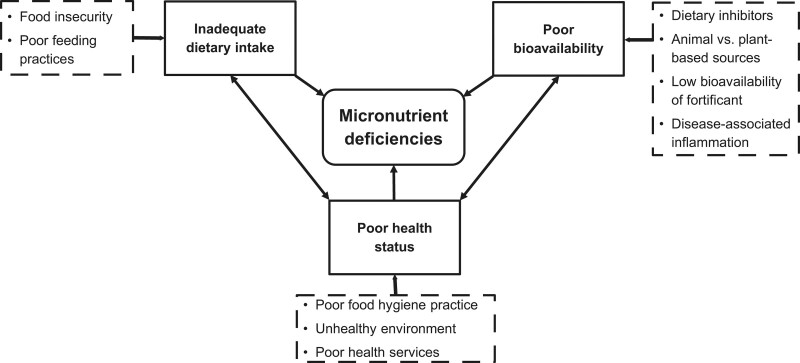
Recommended sequence of introducing complementary food groups across 41 countries in the WHO European region. Modified from reference (24).

The types of complementary foods recommended as first foods varied widely amongst countries. Introduction of animal source, iron-rich foods (such as meat, fish, and eggs) is recommended from 6 months of age in 30 out of 43 countries, however, 13 countries recommended later introduction, some only beginning at 9 months of age (24) (Fig. 3).

Thus, national recommendations vary widely and often differ from WHO recommendations. Differences in global and regional recommendations between the WHO, the European Food Safety Authority, and ESPGHAN may impede harmonization of national recommendations (24). Therefore, periodic reviews and updates of IYCF recommendations are advisable, preferably in close collaboration between governmental bodies, WHO, pediatric societies, and other key stakeholders.

## NUTRITIONAL QUALITY OF COMMERCIAL BABY FOODS IN EUROPE

### João Breda

There is growing concern that some baby foods are of poor nutritional composition and inappropriately promoted. The WHO Regional Office for Europe and Member States devoted attention to this issue to create evidence and provide opportunities for discussion and concerted action.

Some infant and toddler foods resemble adult snacks and do not meet the nutritional needs of infants and young children. A decade ago, research from Canada demonstrated that some baby and toddler food products had high levels of sodium or excessive sugar. This has also recently been reported globally, for example, in studies from South Africa and the USA, baby foods were high in sugar and salt, and were inappropriately promoted (25,26).

In Europe, commercial baby foods contribute a substantial proportion of total dietary energy in infants and young children (22). In 2019, Public Health England issued a report demonstrating that commercial complementary foods contributed significant amounts of total sugar and energy to infants 6–12 months of age. An editorial in the Lancet referenced the report and stated that commercial baby foods were likely fueling childhood obesity and related diseases, and proposed mandatory limits on free sugars in CFD and beverages (27).

In addition, the provision of pureed CFD through baby food pouches may sacrifice valuable learning experiences. Pouches forego opportunities for learning to chew, taste and experience new flavors, textures, and the visual and gustative stimulation provided by whole foods (28). The regular sucking of sweet, semiliquid purees from such pouches may increase the risk of developing dental caries as well as overweight and obesity (28).

The WHO European Childhood Obesity Surveillance Initiative monitors overweight and obesity prevalence in school-aged children (29). In the 36 participating European countries, overweight and obesity prevalence was 29% for boys and 27% for girls aged 7–9 years (29). Early childhood nutrition is a critical pillar to ending childhood obesity and non-communicable diseases which also requires improving nutritional quality of baby foods (30).

Two studies analyzed complementary foods across the WHO European region (31,32). Around a third of products contained sugar or other sweetening agents. A survey of 8000 baby food products in 4 European cities showed >30% of energy from total sugar in 18-57%. Between 13 and 35% of products had food labels claims on child health or development, and up to 60% were marketed as suitable for infants under the age of 6 months.

In a study of 2634 baby foods from ten European countries (33) a third of the energy content came from total sugar. In a separate, cross-sectional survey in 2019 of all commercial baby foods in the UK, nutrient composition was compared to products from 2013 (34) demonstrating no reduction in sweetness, and more snack foods (34). This coincides with observations that economic and political strategies of baby food industries are shaping food systems, and expanding the market on a global scale (35).

## OPPORTUNITIES FOR NUTRITIONAL PROFILING OF BABY FOODS

### Kremlin Wickramansinghe

Nutrient profiling classifies foods according to their nutritional composition, helping to determine which foods have appropriate nutritional composition, and can contribute to promote health and to prevent disease. A nutrient profile model (36) was developed by the WHO Regional Office for Europe to help reduce marketing of foods high in energy, saturated fat, trans fatty acids, sugar, or salt. Only few countries have applied a nutrient profile model to enforce marketing restrictions.

In 2019, a nutrient profile model for commercial baby foods was piloted in ten European countries. The model aims to guide decisions on appropriateness of marketing. It references existing European Commission Directives and Codex Alimentarius (37) food standards, and reflects the approach used for foods marketed for older children in Europe. The model was used to report the nutritional quality of 732 commercially complementary foods in 2 districts in Warsaw, Poland in 2021 (38), and expansion to more European countries, Russia and Central Asia is planned.

Challenges of nutrient profile modeling include: missing nutrient information on food labels, streamlining data across similar products to create nutrient thresholds applicable to all foods and countries, and developing food categories that apply to different types of baby foods.

## PRACTICES OF AND STANDARDS FOR MARKETING OF COMPLEMENTARY FOODS

### Melissa Theurich

Marketing and promotion of CFD to infants and young children includes packaging with bright colors, cartoon characters, toys, images, health claims, or discounts which influence children and their parents to purchase them. Inappropriate marketing and promotion of CFD exists in high, middle- and low- and middle-income countries. A survey of 363 commercial complementary foods products in Taiwan reported 48% of products marketed to infants less than 6 months of age. More than half of the products had high sugar content and 20% had a high sodium content (39). A significantly greater proportion of the products with “no added sugar” claims had high sugar content compared to those without such claims. Generally, products with health claims had higher sodium or sugar contents than those without. Products with calcium or iron content claims did not contain more calcium or iron than products without such claims (39).

On a global level, a survey of 101 countries demonstrated countries are lacking policies on CF counseling, and only few countries (17%–27%) have policies that regulate marketing of CFD (40). The history of the global regulatory environment and standards for marketing complementary foods is important for understanding current global CF policies. Foundational documents on the composition and marketing of commercial CFD include the International Code of Marketing of Breastmilk substitutes (41) and the Codex Alimentarius (37). Global implementation guidance on CF of breastfed and non-breastfed children were published by WHO in 2003 and 2005 (2,42). The WHO global office has announced the ongoing development of global CF guidance (43). In 2017, WHO published 7 recommendations in the Implementation Guidance on ending promotion of inappropriate foods for infants and young children (Table 2).

**TABLE 2.** Summary of recommendations given in the 2017 WHO Implementation Guidance on ending the inappropriate promotion of foods for infants and young children

Recommendation 1	Optimal infant and young child feeding should be promoted based on the guiding principles for complementary feeding and feeding non-breastfed children 6–24 months of age with an emphasis on nutrient-rich, home-prepared, and locally available foods.
Recommendation 2	Products that function as breastmilk substitutes should not be promoted.
Recommendation 3	Foods for infants and young children that are not products that function as breastmilk substitutes should be promoted only if they meet all the relevant national, regional and global standards for composition, safety, quality, and nutrient levels, and are in line with national dietary guidelines.
Recommendation 4	The messages used to promote foods for infants and young children should support optimal feeding and should not include inappropriate messages.
Recommendation 5	There should be no cross-promotion for breastmilk substitutes indirectly via the promotion of foods for infants and young children.
Recommendation 6	Companies that market foods for infants and young children should not create conflicts of interest in health facilities or throughout health systems. Health workers, health systems, health professional associations, and non-governmental organizations should likewise avoid such conflicts of interest.
Recommendation 7	The WHO Set of recommendations on the marketing of foods and non-alcoholic beverages to children should be fully implemented, with particular attention given to ensuring that settings where infants and young children gather are free from all forms of marketing of foods high in fats, sugars, or salt.

## POLICY IMPLICATIONS

### Moderators and All Panelists

Panelists and participants overwhelmingly agreed that many governments have absent, contradictory or lenient policies on marketing practices for commercial CFD. Concerns were raised about the separate global guidance for breastfed and non-breastfed infants, which neglects the many infants who are mixed-fed. There is ongoing scientific discord regarding timing of introduction of CF at global and regional levels. However, there was agreement that focus should shift from the timing of CF alone, to put more emphasis on the quality of both commercial and homemade baby foods, and on ending inappropriate marketing practices. Activities to promote optimal CF practices should include adoption of evidence-informed policies to control inappropriate promotional activities. However, it is also important to provide education for parents on the appropriate composition and preparation of home-prepared foods, as these may be used instead of or combined with commercial complementary foods. Considering the poor complementary practices globally, more concerted and collaborative efforts of the key stakeholders are required at regional and global levels to move CF policies forward. Guidelines should be taken up and implemented by Member States to improve the situation locally.

## CONCLUSIONS

Improving CF practices is an important public health priority. The marked health impact of CF practices is clearly documented in the scientific literature. Meeting micronutrient requirements during the CF period remains a major challenge particularly in disadvantaged, but also in affluent populations. Evidence has accumulated showing that the practices of CF are related to the risk of developing food allergies. Reports from the WHO Regional Office for Europe and others show that the nutritional quality of many commercial baby foods is unsatisfactory, and inappropriate marketing practices are widespread. One opportunity for action is use of nutrition profiling models. There is large variation within Europe on national CF recommendations and many national policies and standards are inadequate. National governments should prioritize policy action on CF, including regulation of inappropriate marketing of CFD. Renewed efforts for collaboration between scientists, public health experts, organizations of pediatricians and other health care professionals, national governments, the WHO, and other stakeholders are recommended to achieve timely progress.
